# The Pupillary Light Reflex as a Biomarker of Concussion

**DOI:** 10.3390/life11101104

**Published:** 2021-10-18

**Authors:** Frederick Robert Carrick, Sergio F. Azzolino, Melissa Hunfalvay, Guido Pagnacco, Elena Oggero, Ryan C. N. D’Arcy, Mahera Abdulrahman, Kiminobu Sugaya

**Affiliations:** 1 College of Medicine, University of Central Florida, Orlando, FL 32816, USA; ksugaya@ucf.edu; 2 Burnett School of Biomedical Science, University of Central Florida, Orlando, FL 32816, USA; 3 MGH Institute for Health Professions, Boston, MA 02129, USA; 4 Centre for Mental Health Research in Association with University of Cambridge, Cambridge CB2 1TN, UK; 5 Carrick Institute, Cape Canaveral, FL 32920, USA; sergio@azzolino.com (S.F.A.); melissa@righteye.com (M.H.); guido@vestibtech.com (G.P.); Elena@vestibtech.com (E.O.); 6 Department of Electrical and Computer Engineering, University of Wyoming, Laramie, WY 82071, USA; 7 BrainNET, Health and Technology District, Vancouver, BC V3V 0C6, Canada; ryan@healthtechconnex.com; 8 Centre for Neurology Studies, HealthTech Connex, Vancouver, BC V3V 0C6, Canada; 9 DM Centre for Brain Health, Department of Radiology, University of British Columbia, Vancouver, BC V6T 1Z4, Canada; 10 Health Informatics and Smart Health Department, Health Regulation Sector, Dubai Health Authority, Dubai 7272, United Arab Emirates; marad@dha.gov.ae

**Keywords:** pupil light reflex, concussion, gender difference, brain function, pupillometry

## Abstract

The size of our pupils changes continuously in response to variations in ambient light levels, a process known as the pupillary light reflex (PLR). The PLR is not a simple reflex as its function is modulated by cognitive brain function and any long-term changes in brain function secondary to injury should cause a change in the parameters of the PLR. We performed a retrospective clinical review of the PLR of our patients using the BrightLamp Reflex iPhone app. The PLR variables of latency, maximum pupil diameter (MaxPD), minimum pupil diameter (MinPD), maximum constriction velocity (MCV), and the 75% recovery time (75% PRT) were associated with significant differences between subjects who had suffered a concussion and those that had not. There were also significant differences in PLR metrics over the life span and between genders and those subjects with and without symptoms. The differences in PLR metrics are modulated not only by concussion history but also by gender and whether or not the person has symptoms associated with a head injury. A concussive injury to the brain is associated with changes in the PLR that persist over the life span, representing biomarkers that might be used in clinical diagnosis, treatment, and decision making.

## 1. Introduction

The size of our pupils changes continuously in response to variations in ambient light levels to regulate the amount of light entering our eyes. This process is known as the pupillary light reflex (PLR) [1]. The PLR, while considered an example of a basic neurological reflex, is not a simple reflex as its function is modulated by cognitive brain function and interaction with the superior colliculus (SC), pretectal olivary nucleus (PON), and locus coeruleus that regulate the PLR [2]. The PLR has probably been observed since the beginning of time and has become a standard examination tool used by all health care professionals after it was first described by Rhazes of Baghdad in the ninth century [3].

It is essential to understand that the PLR is not a linear reaction of the pupil to light because pupil size is dramatically confounded by modest changes in attention, accommodation, and environmental ambient light [4]. The attentional state of an individual, along with the size, luminance, and eccentricity of a stimulus, contributes to what was previously considered a simple reflex [5]. The PLR has been widely used to evaluate the activity of the autonomic nervous system [6]. Interestingly, constriction and dilation of the pupil may be more sensitive than other measures of autonomic function, with constriction dominated by the parasympathetic pathway and pupillary dilation dominated by the sympathetic system [7].

There are gender differences in autonomic function that are important clinically. Interestingly, the iris muscle is modulated by light stimulation activation of the parasympathetic and sympathetic nervous system resulting in higher parasympathetic and lower sympathetic activity in females compared to males, similar to the cardiovascular system [8]. This is due to central processing as a constant amount of light stimulation at a maintained luminance results in a different PLR response for males and females. PLR function is also correlated with other tests of autonomic function. For instance, heart rate variability (HRV) is significantly correlated with PLR variables for evaluating the activity of the autonomic nervous system with the most robust measures of parasympathetic activity [9].

The PLR represents a complexity of integration at many brain, brainstem, and spinal cord levels such that it should not be considered as a simple response to a light source stimulation. There is, therefore, a need to understand the neurophysiology associated with brain function that might modulate the PLR. When light enters the eye, it is immediately converted to electrical impulses by specialized retinal rod and cone cells in the neuroretina [10,11,12]. These impulses are integrated by six types of neuronal cells in a layered functional unit of photoreceptors and glial cells that form the optic nerve [13]. The nasal retinal fibers of each eye contribute to the optic nerve and cross at the optic chiasm joining temporal retinal fibers from the other eye where they continue as the optic tract to the visual cortex of the occipital lobe at the back of the brain [14]. The optic tract gives off fibers that synapse and excite the mesencephalic olivary pretectal nucleus (OPN) [15,16], which is excited by light from the retina but is also modulated by output from the cerebral cortex [2]. OPN discharge results in bilateral activation of the Edinger Westphal nucleus (EWN), a preganglionic parasympathetic pupil constricting center [17,18]. While the OPN excites the EWN, the locus coeruleus (LC), the principal synthesizer of norepinephrine, inhibits the EWN. Its output is the result of a combination of excitation and inhibition [19,20,21,22].

The resultant discharge rate of the EWN results in pupil constriction because of its output and excitation of the ciliary ganglion (CG) that excites the pupiloconstrictor muscle [18,23]. The EWN excitation of the CG results from mathematical modeling of the direct activation of the EWN by the OPN and its direct inhibition by the LC [24]. However, the LC output is modulated by multisynaptic activators from the brain and brainstem that affect the amount of pupil constriction via its effect on the EWN [25]. A crucial clinical understanding of function is that pupil constriction is constantly changing in response to retinal activation by a light source. However, at the same time, the PLR is under the influence of other areas of the nervous system that are independent of the light source [22,26]. As such, the function of the brain affects or modulates the size of the pupil and its reaction to light, even though many of the structures that influence the PLR are not associated with the light-mediated PLR. For instance, cognitive processes that are often impaired after traumatic brain injuries are central to the prefrontal cortex (PFC) function, which modulates the PLR [27].

Brain injury changes affect the PLR latency of its activation, pupil size, the velocity of constriction and dilation, and recovery times. The brain excites the paraventricular nucleus (PVN), which excites the sympathetic intermediolateral cell nucleus (IML) in the spinal cord [28]. This cascade of events is such that the IML activates the suprachiasmatic nucleus (SCN), which activates the pupillodilator muscle (PD), causing pupil dilation. At the same time, the IML also receives excitation from the LC, which is itself excited by the cerebral cortex [29]. From a functional viewpoint, cortical lesions of function or pathology may affect both parasympathetic pupillary constriction via a change in activation of the LC, resulting in changes of EWN inhibition and sympathetic pupillary dilation via IML activation. 

All of this activity is happening at the same time such that the central role of the brain in modulating the PLR is further confounded because the LC is also activated by the dorsomedial hypothalamus (DMH), which is activated by the cerebral cortex, affecting both parasympathetic and sympathetic influences of the PLR [2]. While functional brain connectivity drives the integration of target areas involved in the PLR, light activation of the retina is associated with direct activation of the OPN and the SCN that excites the DMH while inhibiting the PVN excitation of the IML. At the same time, the DMH is activated by the cerebral cortex that also activates the DMH target (LC) that excites the IML. While the DMH can be considered a part of the sympathetic pupil constriction pathway to the IML because it activates the LC excitation of the IML, it is also under the influence of the SCN that excites it while at the same time inhibiting the PVN activation of the IML [30,31].

**Figure 1:**
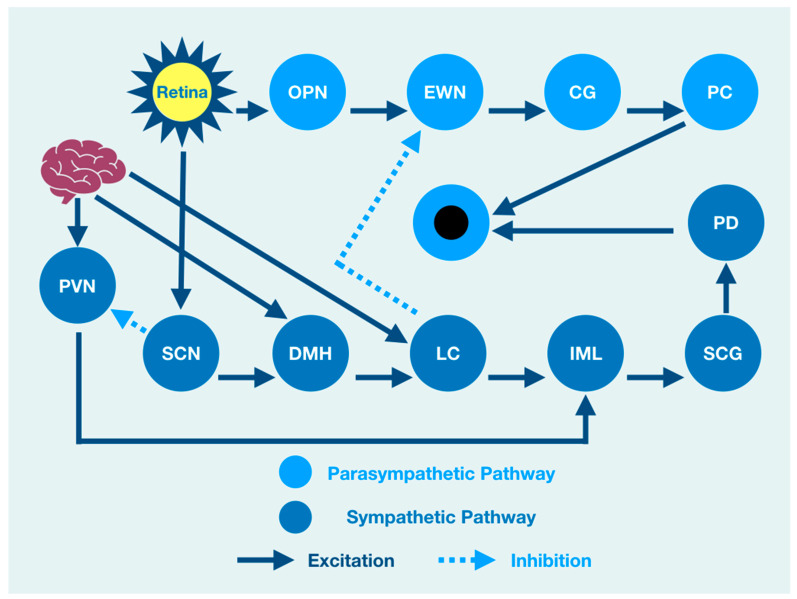
The integrated PLR. Sympathetic Pathway: SCN: suprachiasmatic nucleus, DMH: dorsomedial hypothalamus, LC: locus coeruleus, IML: intermediolateral column, SCG: superior cervical ganglia, PD: pupil dilator muscle. Parasympathetic Pathway: OPN: olivary pretectal nucleus, EWN: Edinger Westphal nucleus, CG: ciliary ganglion, PC: pupil constrictor muscle, PVN: paraventricular nucleus.

An overview cartoon of the integrated PLR demonstrates the functional clinical integration of brain, brainstem, and spinal cord structures that contribute to pupil size after light stimulation (Figure 1).

The PLR represents much more than a simple neurological reflex. Pupillometry represents a diagnostic technology that provides a reliable quantitative evaluation of the PLR that is reproducible [32]. Reproducibility of a diagnostic test is necessary if clinicians desire to use the test to quantify function over time. Fortunately, the assessment of pupil function has been demonstrated to be reliable and vital in the evaluation of all patients, including neurocritical patients [33]. The value of the PLR is, therefore, more than a simple quantification of a reflex. Even acute brain-injured patients will benefit from quantitative pupillometry for various brain functions, including delirium [34].

The global incidence of traumatic brain injury (TBI) represents 64–74 million cases annually, resulting in one of the world’s most significant incidences of morbidity and mortality [35]. We wondered whether people that have had a concussion with and without symptoms might have functional brain changes that the PLR might measure. As the PLR is modulated by central brain function, any long-term changes in brain function secondary to injury should cause a change in the parameters of the PLR compared to non-concussion subjects. We hypothesized that there would be differences in PLR components between concussion and non-concussion subjects that also would manifest in both gender and age effects. 

## 2. Methodology

We performed a retrospective clinical review of the PLR of patients attended in neurological clinics in our network over the calendar year 1 January 2019 to 1 January 2020 using the BrightLamp Reflex iPhone app. All PLR recordings included data based on the Minimum Information about a Neuroscience Investigation (MINI), published by the CARMEN consortium adapted for pupillography as initiated at the 32nd International Pupil Colloquium 2017 in Morges, Switzerland [36]. The PLR recordings were obtained only at the initial presentation and we did not include PLR recordings obtained after the initial examinations. All subjects were classified as having suffered a concussion if their history documented a concussion diagnosis by a health care professional and as having no concussion if they stated that they had never experienced a concussion or had received a diagnosis of having had one. Patients reported as having a history of mental illness or brain surgery were not included in the analysis. All data were obtained using the BrightLamp iPhone Reflex App to obtain 5-s PLR data from subjects that had suffered a concussion and those that had not. Reflex’s FDA registration number is 3015176913, with a device listing number of D334435. Reflex is a Class 1 510(k) exempt medical device. The Reflex App is an iPhone clinical tool that records a video of a subject’s eye at 30 Hz, which is parsed into images and is passed into a trained object detector that allows for rapid identification of an iris. The iPhone Reflex App creates a photic flash stimulation of approximately 1 ms. in duration at 50 lumens set at 100% power (flash brightness), capturing dynamic responses at 30 Hz or 30 frames each second. The iPhone was held by the examiner who visualized the pupil and initiated the flash stimulation by pressing the camera button. All subjects observed a minimum of 60 s of rest before obtaining the PLR and rested a minimum of 60 s between each test. We measured the PLR variables of latency, maximum pupil diameter, minimum pupil diameter, maximum constriction velocity (MCV), and the 75% recovery time (75% PRT). 

### Statistical Analysis

All analysis was done in STATA 17 (Stata Corp, College Station, TX, USA). Statistical analysis included calculating means and standard deviations for each PLR variable for both normal and concussion patient groups that were further grouped by gender, age, and the presence or absence of concussion symptoms. We used multiple linear regression models as well as two-sample t-tests with equal variances between all variables.

## 3. Results

### 3.1. Summary Statistics

**Figure 2:**
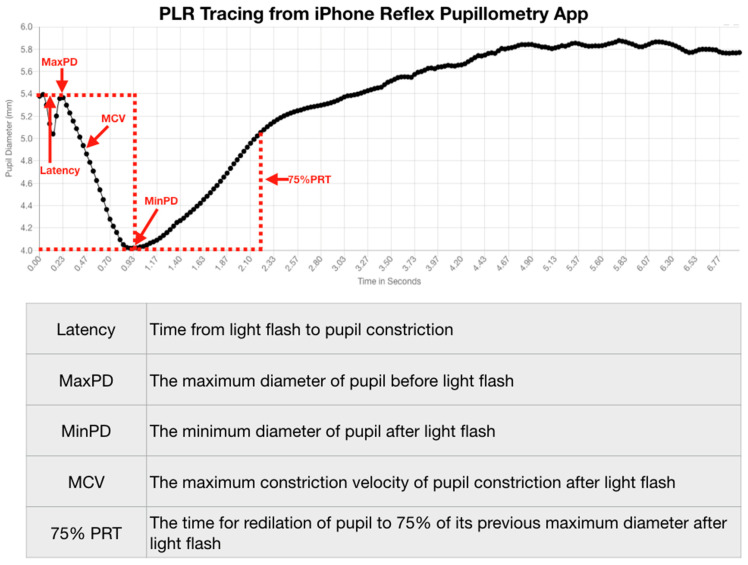
PLR Tracing from iPhone Reflex Pupillometry App.

We stratified our data by identifying the pupillometry metrics that quantify the PLR and their relationships to concussion and symptom status. We measured the PLR variables of latency, maximum pupil diameter, minimum pupil diameter, maximum constriction velocity (MCV), and the 75% recovery time (75% PRT) (Figure 2).

We then classified the variables as to age and gender, whether or not the subject had suffered a concussion, and whether or not they had symptoms associated with a concussion at the time of the examination. The 75% PRT was obtained only from those subjects that had suffered a concussion. The study demographics are summarized in Table 1. 

### 3.2. Summary of Age, Gender and Concussion by Decade

We also divided subjects’ ages into blocks by decades and categorized them as to gender and concussion (Table 2).

### 3.3. Maximum Pupillary Diameter (MPD)

There was no significant difference between the MPD in males and females who did not suffer a concussion and had no concussion-like symptoms. Both males and females with no history of concussion but experienced concussion-like symptoms had highly significant differences between their MPD and people without a history of concussion or concussion-like symptoms. Both males and females with a history of concussion, regardless of whether or not they had a present or history of symptoms, had highly significant differences between their maximum pupil diameter compared to people who had never had a concussion (Table 3). 

**Figure 3:**
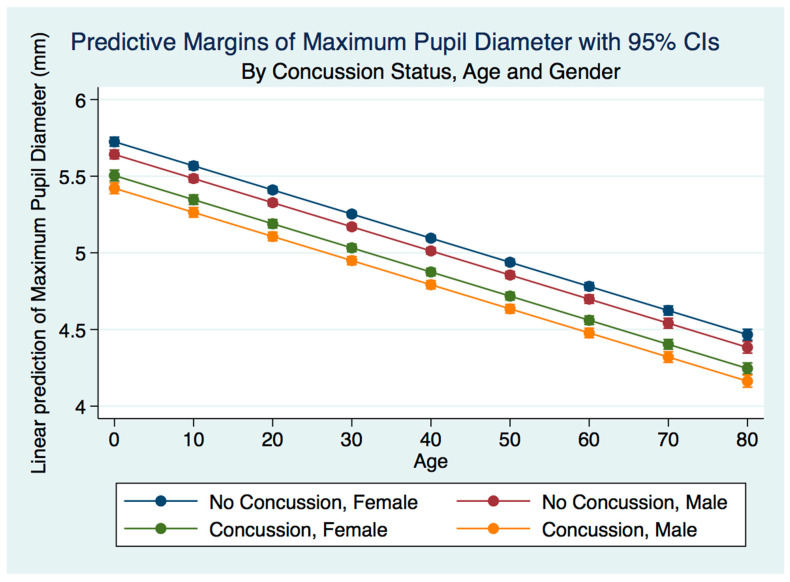
Predictive Margins of Maximum Pupil Diameter by Concussion Status, Age, and Gender.

A multiple linear regression model predicting the MPD by gender, age, and concussion status was statistically significant (*p* = 0.0000, R^2^ = 0.0834). The maximum pupil diameter for both males and females was smaller for both males and females after concussion compared to the non-concussion group. The males who had suffered a concussion had a smaller maximum pupil diameter than the females. The non-concussion group was associated with a smaller maximum pupil diameter for males compared to females. As the age of subjects increased, the maximum pupil diameter decreased for both genders for both the concussion and non-concussion groups (Figure 3).

### 3.4. MPD over Time after Concussion

**Figure 4:**
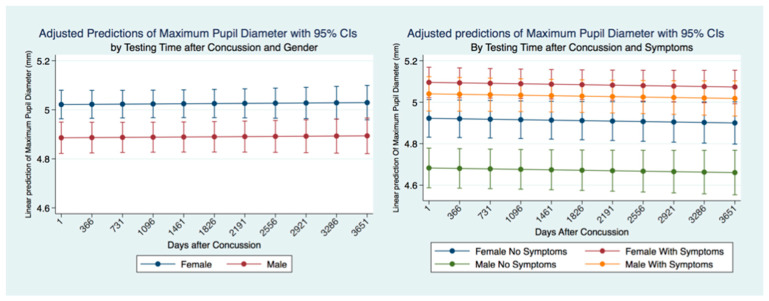
MPD for concussion subjects by gender and symptoms over ten years post-concussion.

We wanted to see if the MPD was stable over time and if there were differences between genders and for individuals who had suffered concussions and had symptomatology. A linear regression model predicting the MPD for ten years revealed that the MPD was relatively stable over time. Males had a significantly smaller MPD than females, and this difference was maintained over ten years after a concussion. We controlled for symptoms and gender in concussion subjects over time. We found that females with symptoms had the largest MPD, significantly higher than females without symptoms and males with and without symptoms. Males with symptoms had a significantly larger MPD than males without symptoms who demonstrated a larger MPD than females without symptoms and males without symptoms. Both males and females with symptoms had a larger MPD than those without symptoms. This relationship was maintained over the ten years following a concussion with a minor non-significant trend to decrease the size of the MPD over time (Figure 4).

### 3.5. Minimum Pupillary Diameter (MinPD)

The MinPD for all normal healthy subjects (male and female combined) was 33.60 mm. Subjects in our data bank that included metrics grouped by gender, concussion, and symptomatology class had a significantly larger MinPD than the general population (did not constrict as much to light) (Table 4). 

**Figure 5:**
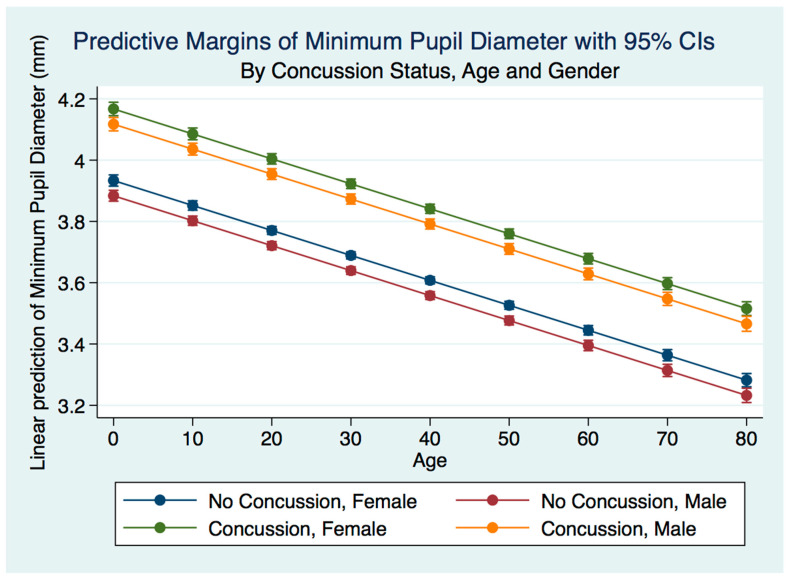
MinPD for concussion subjects by gender and age.

A multiple linear regression model predicting the minimum pupil diameter by gender, age, and concussion status were statistically significant (*p* = 0.0000, R^2^ = 0.0675). The minimum pupil diameter for both males and females was larger for both males and females after concussion compared to the non-concussion group (*p* = 0.0000). The males that had suffered a concussion had a smaller minimum pupil diameter than the females, and the non-concussion group was associated with a smaller minimum pupil diameter for males compared to females. As the age of subjects with a concussion increased, the minimum pupil diameter decreased for both genders for both the concussion and non-concussion groups. The spread between the minimum pupil diameter between concussion and non-concussion subjects was far more pronounced than the spread between the maximum pupil diameter between the same groups (*p* = 0.000) (Figure 5).

**Figure 6:**
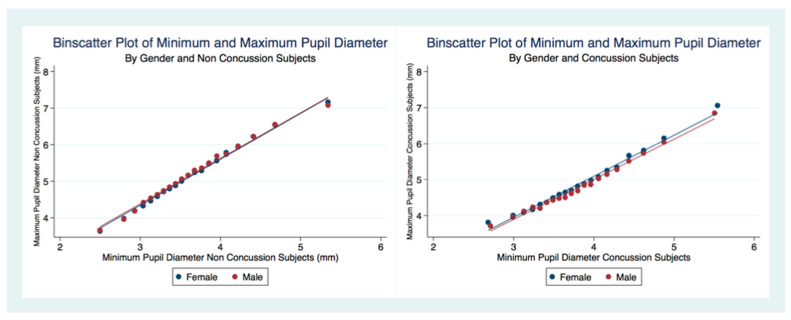
Relationship between MPD and MinPD by concussion status.

There was a linear relationship between the MPD and the MinPD regardless of concussion status or gender (Figure 6). A larger MPD was associated with a larger MinPD. The concussion subjects had a decreased range of pupil constriction (MPD–MinPD) compared to non-concussion subjects. 

### 3.6. Pupillary Recovery Time (75%)

**Figure 7:**
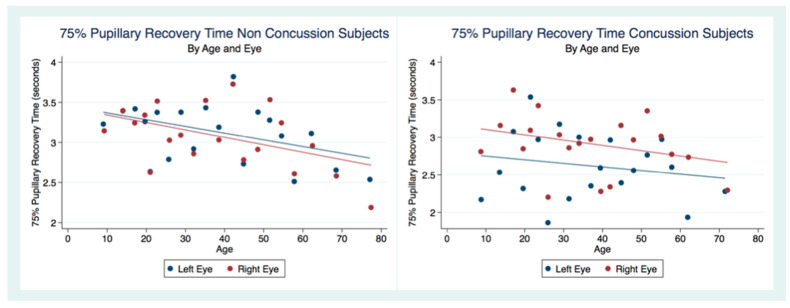
The 75% Pupillary Recovery Time by Age, Eye, and Concussion Status.

We considered the subjects that had not suffered a concussion as controls to compare the 75% pupillary recovery time (75% PRT) of concussion subjects. We had 3211 left eye 75% PRT and 3870 right eye 75% PRT obtained from subjects that had never experienced a concussion. The concussion group had 991 left eye 75% PRT and 1108 right eye 75% PRT. Both eyes in the concussion group had a 75% PRT that was significantly faster than the non-concussion group. In order to discern patterns in this large sample, we created scatterplots by dividing the independent variables into a series of 20 bins and calculated the mean values for all points in each bin. We placed a dot for the mean values on the outcome variable. Each scatterplot in our analysis was created in this fashion (Figure 7). 

A two sample unpaired t test comparing differences between the right eye 75% PRT between concussion and non-concussion groups was statistically significant (*t* (4976) = 2.0001, *p* = 0.0455, 95% CI (0.0030376, 0.3034155)) as were the left eye 75% PRT between these groups (*t* (4200) = 5.9314, *p* = 0.0000, 95% CI (0.3337997, 0.6634134)) (Table 2). A two sample unpaired t test comparing differences between the left eye 75% PRT and the right eye 75% PRT of the 7081 75% PRTs from non-concussed subjects was not statistically significant (*t* (7079) = −0.7988, *p* = 0.4244, 95% CI (3.042349, 3.148847)) (Table 2) and we therefore combined the 75% PRT from both eyes for all analysis in this investigation. 

**Figure 8:**
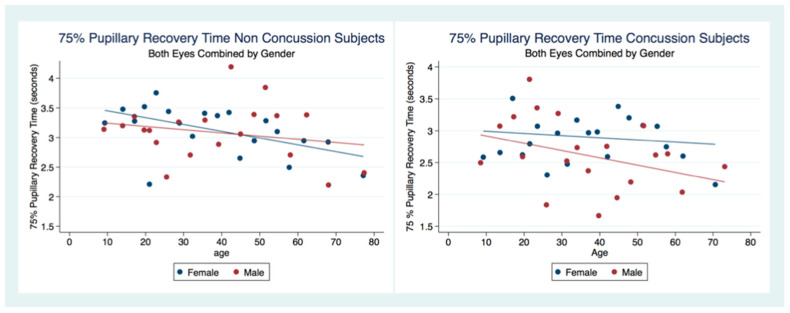
The 75% Pupillary Recovery Time by Age, Gender, and Concussion Status.

There was no statistical difference between the 75% PRT between males and females that had not suffered a concussion (*t* (6949) = 0.3522, *p* = 0.7045, 95% CI (3.053171, 3.161027)). However, there was a statistical difference between genders for those subjects that had been concussed (*t* (2097) = 2.5544, *p* = 0.0107), 95% CI (2.683494, 2.876747) (Table 2). The 75% PRT for both genders combined were significantly faster for the concussed group (*t* (9178) = −5.5692, *p* = 0.0000, 95% CI (2.976755, 3.070173)) with male and female concussion subjects demonstrating faster 75% PRT than those for both genders that did not have a concussion (Males *t* (3765) = −5.3244, *p* = 0.0000), 95% CI (2.906674, 3.052012), Females *t* (5281) = −2.8622, *p* = 0.0042, 95% CI (3.006275, 3.130289) (Table 2). In the non-concussion group, the males had a quicker 75% PRT than females up to approximately 45 years of age when the 75% PRT for females became faster than that of males. In the concussed group, males had a faster 75% PRT throughout the lifespan with a steady and significant increase in speed of 75% PRT as age increased (Figure 8).

**Figure 9:**
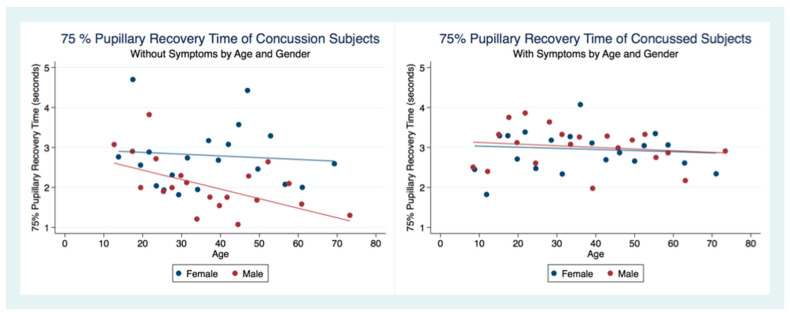
The 75% Pupillary Recovery Time of Concussion Subjects by Symptoms, Age, and Gender.

We were interested to see if there were any differences in the 75% PRT between those concussion subjects with symptoms and those who did not. Of those subjects that had suffered a concussion, 1277.75% PRT represented subjects with symptoms, and 822.75% PRT were obtained from concussion subjects that no longer had symptoms. A 2 sample unpaired t-test for differences between the 75% PRT of concussion subjects that had symptoms compared to those without symptoms was highly statistically significant (*t* (2097) = 5.3005, *p* = 0.0000, 95% CI (2.683494, 2.876747)) (Table 5). The symptomatic concussion group had a slower 75% PRT for both male and female subjects than the non-concussion group without a significant difference between genders. Although the asymptomatic concussion group had faster 75% PRT, there was a significant gender bias, with males having much quicker 75% PRT than females, which was increasingly faster as age advanced. The women in both groups had relatively stable 75% PRT over the age spans, maintaining a faster 75% PRT in the women with asymptomatic concussions vs. those with symptoms (Figure 9).

**Figure 10:**
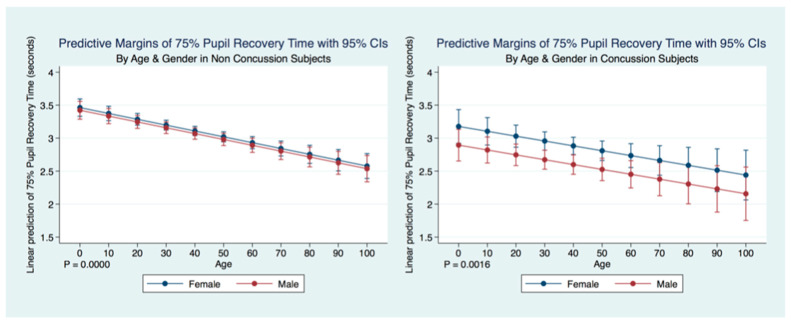
Differences in Predictive Margins of 75% Pupil Recovery time by Age and Gender in Non-Concussion and Concussion Subjects.

A multiple regression model to predict the Non-Concussion subjects 75% PRT by age and sex was statistically significant (F (26,943) = 18.40, *p* = 0.0000, R^2^ = 0.0053) as was a multiple regression model to predict Concussion subjects 75% PRT by age and sex (F (2, 2095) = 6.43, *p* = 0.0016, R^2^ = 0.0061). Interestingly, in both Concussion and Non-Concussion subjects, the 75% PRT becomes increasingly faster over the lifespan. The Concussion subjects mirroring the age trends as a predictor of 75% PRT, but with an even quicker response. There is a gender bias for subjects that have suffered a concussion as males have a significantly quicker 75% PRT than females, while the non-concussion group did not have a gender bias (Table 5) (Figure 10).

**Figure 11:**
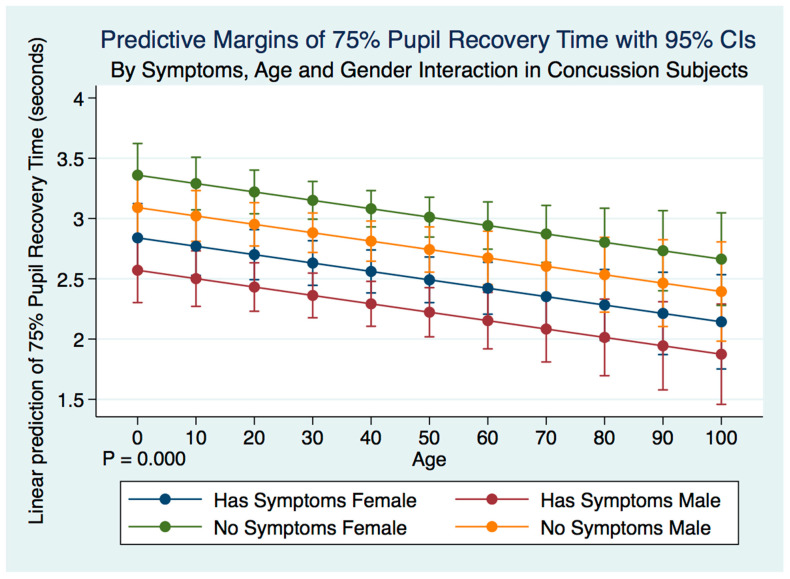
Differences in Predictive Margins of 75% Pupil Recovery time by Symptoms, Age, and Gender in Concussion Subjects.

A multiple regression model predicting the 75% PRT of concussion subjects by symptoms, age, gender, and their interactions was highly statistically significant (F (5, 2092) = 11.60, *p* = 0.0000, 95% CI (2.863738, 3.727156) (Figure 2). Both male and female subjects who no longer had symptoms after a history of a concussion demonstrated a significantly slower 75% PRT than those who maintained symptoms after a concussion. Females with symptoms had a slower 75% PRT than males with symptoms, and females without symptoms had a slower 75% PRT than males without symptoms. The symptomatic grouping of both males and females had a faster 75% PRT than did the grouping of both males and females without symptoms (Table 5) (Figure 11).

**Figure 12:**
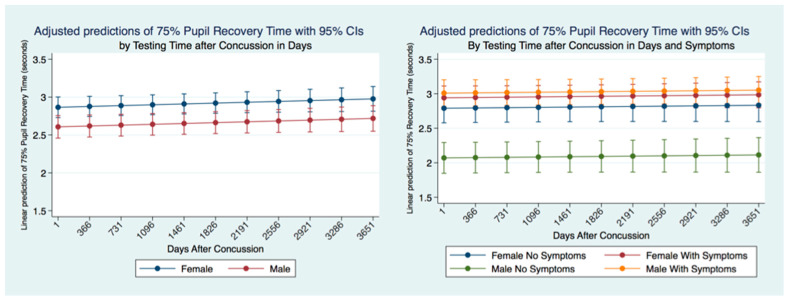
Adjusted Prediction of 75% Pupil Recovery Time by Gender, Concussion, and Symptom Status.

We wanted to see whether the 75% PRT was stable up to 10 years after a concussion or if it would change. We identified the time between the PLR quantification and the time of a reported concussion in all subjects that had suffered a concussion. Females had a significantly slower (*p* = 0.0000) 75% PRT than males maintained over ten years after a concussion with a non-significant gradual slowing over time common to both genders. We controlled for symptomatology and concussion, and we found that males with symptoms had the slowest 75% PRT times followed by females with symptoms. The difference between males and females 75% PRT with symptoms was not significant. However, there was a significant difference (*p* = 0.0000) between the 75% PRT males with and without symptoms. Males without symptoms had the fastest 75% PRT that was significantly different (*p* = 0.0000) than the 75% PRT recovery times for all other classes of subjects. Females without symptoms had a significantly faster 75% PRT (*p* = 0.0000) than females with symptoms. Nevertheless, there was an extremely high significant difference (*p* = 0.0000) in 75% PRT between males and females with symptoms with the males being markedly quicker (Figure 12).

### 3.7. Maximum Constriction Velocity (MCV)

**Figure 13:**
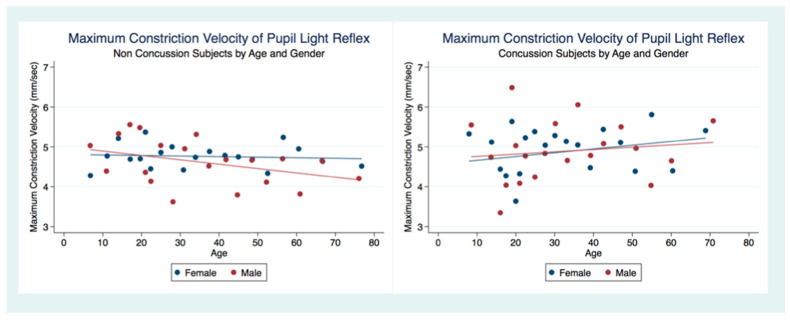
Maximum Constriction Velocity of Pupil Light Reflex by Concussion, Age, and Gender.

MCVs were obtained from 4999 subjects (2767 female, 2232 male) that had suffered a concussion and 22,847 (12,803 female, 10,044 male) that had not. A two-sample unpaired *t*-test for differences between the MCV for both groups revealed that the non-concussion group was slower than the concussion group with statistical significance (*t* (28,673) = −2.7378, *p* = 0.0062, 95% CI (4.64121, 4.760105)). However, the concussion group under the age of 20 demonstrated slower MCVs than the non-concussion group (Table 6) (Figure 13). 

**Figure 14:**
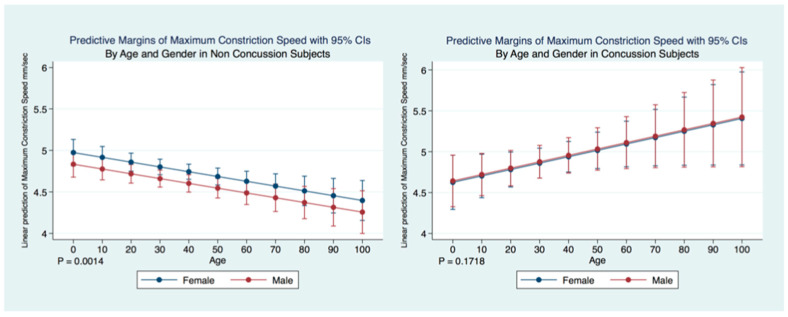
Differences in Predictive Margins of Maximum Constriction Velocity by Age and Gender in Concussion and Non-Concussion Subjects.

A multiple regression model predicting the maximum constriction velocity (MCV) of non-concussion subjects by age and gender was statistically significant (F (5, 22,807) = 6.58, *p* = 0.0014, 95% CI for gender (−0.2771672, −0.0034024) and age (−0.0092983, −0.0022366). Females without a history of concussion had a quicker MCV than did males without a concussion history. The MCV of non-concussion subjects decreased significantly (*p* = 0.000) over the lifespan. A multiple regression model predicting the MCV of concussion subjects by age and gender was not statistically significant (F (2.4991) = 1.76, *p* = 0.1718, 95% CI for gender (−0.2544719, 0.2885153) and age (−0.0003619, 0.0159813) and there were no significant differences observed between the MCV of males and females that had suffered concussions. There was a paradoxical increase in MCV of concussion subjects over the lifespan compared to the non-concussion subjects whose MCV decreased significantly (*p* = 0.0000) (Figure 14).

**Figure 15:**
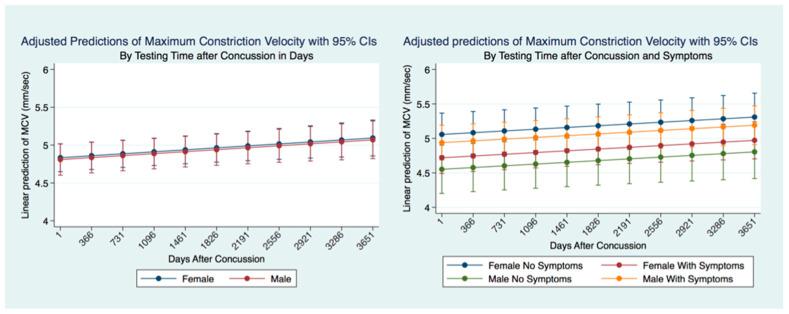
Adjusted Prediction of Maximum Constriction Velocity in Concussion Subjects by Time and Symptoms.

There was no significant difference between males and females that had suffered a concussion over the ten years following the concussion. However, if we consider the presence or absence of symptoms after a concussion, it is clear that there are both gender and significant symptomatology differences (*p* = 0.0000). Females without symptoms after concussion have the fastest MCV, significantly faster than females with symptoms and males with and without symptoms. Males with symptoms had a significantly (*p* = 0.000) quicker MCV than males without symptoms or females without symptoms. The slowest MCV was associated with males without symptoms. Both males and females without symptoms had a slower MCV (Figure 15).

**Figure 16:**
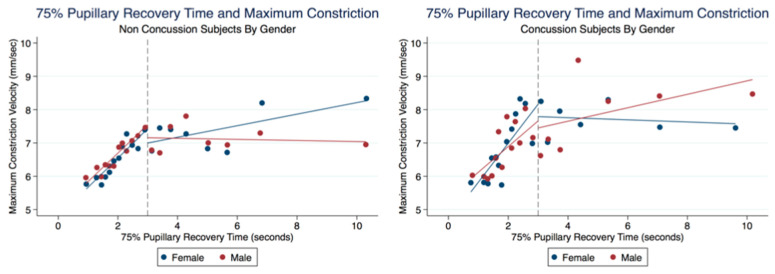
Relationship of MCVs and 75% Pupillary Recovery Time by Age, Gender, and Concussion Status.

There was no significant difference between the MCVs of concussion subjects with and without symptoms (*t* (5000) = −0.0450, *p* = 0.9641, 95% CI (4.747533, 5.015023)) (Table 6). We wanted to explore a relationship between the 75% RCT and the MCV. In both the Concussion and Non-Concussion groups, the 75% PRT and MCVs had a steep linear relationship when the 75% RCT was below 3 s. Subjects in the Concussion group were found to have higher MCV values than Non-Concussion subjects at these quicker 75% RCT. Interestingly, females demonstrated faster MCVs than males when the 75% RCT took longer than 3 s in the Non-Concussion group and slower MCVs than Males in the Concussion group (Figure 16).

**Figure 17:**
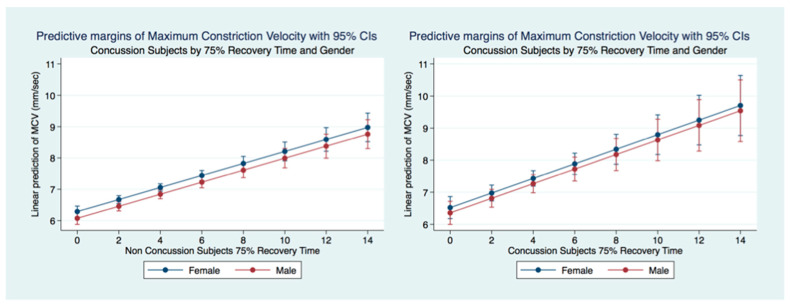
Predictive Margins of MCV by 75% RT, Gender, and Concussion Status.

A multiple regression model predicting the maximum constriction velocity (MCV) of concussion subjects (2767 females and 2232 males) by 75% RT, gender and age was statistically significant (F (3, 1881) = 17.72, *p* = 0.0000, 95% CI (0.1443846, 0.3104978). There was no significant difference between gender for MCV prediction by 75% RT (*p* = 0.9124). A multiple regression model of MCV prediction by 75% in the non-concussion group (12,803 females and 10,044 males) was also highly significant (F (3, 6432) = 40.10, *p* = 0.0000, 95% CI (0.1507661, 0.2330021). The 75% PRT was associated with faster MCV in the Concussion subjects (Figure 17).

### 3.8. Latency of the Pupillary Light Response

Summary statistics for the latency of the PLR are shown in (Table 7).

The latency of the PLR for those subjects with a history of concussion, with or without symptoms, was slower than subjects without a history of concussion (*p* = 0.0000).

**Figure 18:**
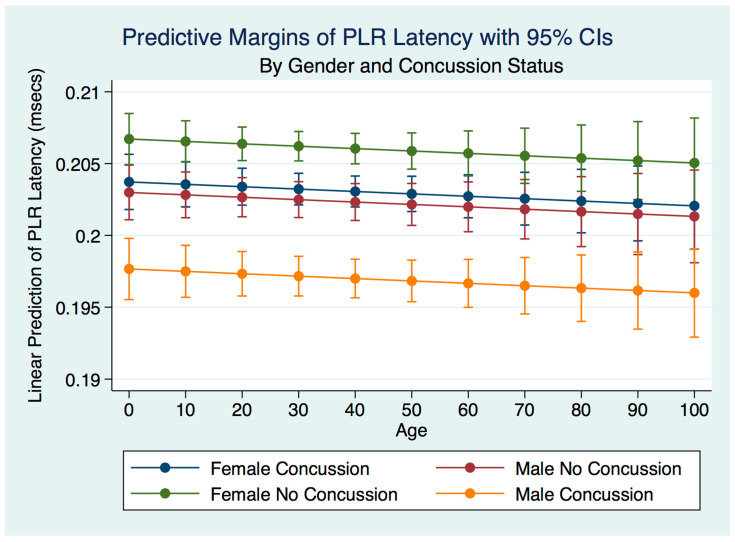
Predictive Margins of PLR Latency by Gender and Concussion Status.

A multiple regression model predicting the latency of the PLR by gender, concussion, and symptom status was statistically significant for prediction by concussion status (F (3, 19,060) = 38.22, R^2^ = 0.0060, *p* = 0.0000). There was a trend toward decreasing the latency over the lifespan. Females who had never experienced a concussion had the fastest latencies, followed by females who had suffered a concussion, with the difference between them statistically significant (*p* = 0.0000). The latencies of males with no history of concussion were slightly slower than females who had a concussion but significantly faster than males who had concussions (Figure 18).

## 4. Discussion

This investigation has provided information about the pupil’s reaction to light in subjects that have suffered a concussion compared to those that have not. The differences between subjects in all metrics of the PLR after concussion contribute to a greater understanding of the functional integration of the brain when it is injured. These functional differences are pronounced when considering the PLR function between genders. While most subjects that suffer a concussion will become symptom-free in a relatively short period [37], it is clear from our findings that the lack of symptoms after a concussion does not represent a return to typical PLR metrics. This is alarming, as it appears that functional brain states in individuals who have suffered a concussion might be similar to those associated with other neurodegenerative disorders such as Alzheimer’s and Parkinson’s disease [38]. These observations also suggest that the PLR might be used as a diagnostic biomarker of the success or failure of treatment strategies. 

The PLR is often considered a simple reflex representative of a quantum of light evoking Cartesian pathways, resulting in a change of pupil diameter. As such, clinicians have been concerned that changes in ambient light observed in different settings might make a baseline measurement of pupil size impossible in anything but laboratory settings that might control for environmental light. However, our findings suggest that light is but one of many integration variables that govern the pupil’s size. Even the magnitude of the PLR in a talking parrot, while affected by light, is modulated more by the attention required for vocalization or recognition of known words by humans [39].

Our findings show that the variables associated with brain function that modulates the PLR might, as in the parrot, be more critical in pupillary functional reaction to light than the light source. The differences in the PLR after concussion support this, and we were able to observe trends in changes of PLR metrics in non-laboratory controlled ambient lighting situations that mirrored the observations of others in a controlled laboratory setting [40]. The reality of this situation translates to the ability to use the PLR in non-traditional settings such as on the sporting field, in the gym, clinic, or hospital wards without great concern for the control of ambient environmental light. 

We have demonstrated that the PLR is different between genders, and that it changes with age. Fortunately, normative age-related data have been calculated for the PLR and can be used as a baseline for patient evaluation [40]. We demonstrated that head injuries affect the PLR differently across age groups, complementing the work of others that have noted that adolescents demonstrate visual disorders with autonomic concomitants that are easily identified by the PLR after concussion [41]. The complexity of cognitive disorders and neurodevelopmental syndromes also have identifiable PLR functions. Patients with attention deficit disorder (ADD) have a greater pupillary diameter and a faster maximum constriction velocity than normal age-matched controls and older subjects [42]. Our findings have delineated the changes in pupillary diameter and constriction velocity controlled by gender, age and the presence or absence of concussion symptoms. We have also demonstrated that the latency of the PLR may serve as a sensitive biomarker that also changes with age. For example, the time delay between a light stimulation and the latency of the PLR is increased in older subjects (46–78 years) compared to younger subjects (18–45 years old) [43]. We have demonstrated that brain injury changes affect the PLR latency of its activation, pupil size, the velocity of constriction and dilation, and recovery times. 

Advances in technology have improved the accuracy of diagnostic instruments and the portability and ease of their use. We have demonstrated that the size of the pupil and its reaction to light can be reliably and reproducibly quantified by an advanced portable instrument using an iPhone medical app for clinical use [44,45]. Furthermore, this quantitative pupillometry has provided us with immediate evaluation and management of traumatic brain-injured patients over time and should contribute to decreasing the associated morbidity and mortality of these injuries [46]. We have provided reliable biomarkers that identify athletes with a concussion because of their larger maximum and minimum pupil diameter compared to healthy controls [47]. Most importantly we have quantified the long-term metrics of the PLR in individuals that have suffered concussions. Most people that suffer from a concussion have a resolution of their symptoms within a short time of days to weeks. At the same time, a smaller number maintain their symptoms and are classified as suffering from post-concussion syndrome (PCS) [48]. We have demonstrated the apparent permanency of functional changes of the PLR after concussion. These life-long changes in neurological function are supported by recent advances in quantification of increased ventricular size and decreased white matter measurements apparent later in life after a concussion [49].

## 5. Conclusions

The PLR is more than a simple reflexogenic change in the size of the pupil when exposed to light. Pupil size is constantly changing and represents a complex modulation of the autonomic nervous system by the functional interaction of the brain that can be measured and quantified by a novel portable pupillometer. Subjects who have suffered a concussion will have different latencies to constriction after light stimulation and different maximum and minimum pupillary diameters. They will also have differences in the speed of contraction and dilation of the pupil after light stimulation. The differences in PLR metrics are modulated not only by concussion history but also by gender and whether or not the person has symptoms associated with a head injury. A concussive injury to the brain is associated with changes in the PLR that persist over the life span, representing biomarkers that might be used in clinical diagnosis, treatment, and decision making. Advances in technology have promoted the ability to quantify biomedical metrics. The metrics obtained from the PLR can assist in determining whether a patient has suffered a concussion regardless of symptomatology. As a physiological measurement, the PLR is not affected by subjective interpretation and might be incorporated as a valuable biomarker of brain functional status. 

## 6. Limitations

This is a retrospective clinical review of PLR quantification of real patients attended in the neurological clinic. It does not have the benefits seen in a prospective randomized controlled trial. As such, a variety of unknown confounders of the data such as unknown or undiagnosed mental illness or disease and the wide and potentially subjective criteria involved in the diagnosis of a concussion that might affect the PLR may be included and affect the data. The large number of patients in this study should mitigate such confounding but cannot eliminate them. 

## Figures and Tables

**Table 1:** Summary statistics of concussion metrics classified by gender, concussion, and symptom status.

	Gender							
	Female				Male			
	Concussion				Concussion			
	No Concussion		Concussion		No Concussion		Concussion	
	Symptoms		Symptoms		Symptoms		Symptoms	
	No	Yes	No	Yes	No	Yes	No	Yes
Frequency	1595.00	5730.00	1079.00	3449.00	1080.00	4641.00	733.00	2325.00
Percent	7.73%	27.77%	5.23%	16.72%	5.23%	22.49%	3.55%	11.27%
**Age**								
Mean	35.15	34.67	40.21	38.17	27.90	30.93	37.44	35.77
SD	20.47	18.36	17.25	17.78	18.58	17.77	17.68	19.04
**Latency**								
Mean	0.21	0.21	0.20	0.20	0.20	0.21	0.20	0.20
SD	0.06	0.06	0.06	0.06	0.06	0.06	0.06	0.06
**Max Diam**								
Mean	5.14	5.14	4.84	4.96	5.03	5.18	4.68	4.89
SD	1.17	1.12	0.83	1.00	1.15	1.12	0.79	0.95
**Min Diam**								
Mean	3.61	3.66	3.89	3.87	3.56	3.64	3.83	3.89
SD	0.64	0.69	0.63	0.69	0.63	0.64	0.61	0.68
**MCV**								
Mean	3.81	3.32	7.23	6.93	3.26	3.66	6.58	7.11
SD	5.40	4.50	3.86	3.93	4.02	5.06	3.60	4.00
**75% PRT**								
Mean			2.98	3.11			2.45	3.00
SD			2.48	2.28			2.13	2.23

**Table 2:** Age of subjects by concussion, decade, and gender.

Concussion			No Concussion	
	Female	Male	Female	Male
Age 1–10 years *n* = 1903				
Mean Age	7.9	7.8	7.2	7.5
Minimum Age	1.0	3.0	1.0	1.0
Maximum Age	10.0	10.0	10.0	10.0
Age 10–20 years *n* = 6257				
Mean Age	16.2	16.5	16.6	15.7
Minimum Age	11.0	11.0	11.0	11.0
Maximum Age	20.0	20.0	20.0	20.0
Age 20–30 years *n* = 6365				
Mean Age	25.0	24.7	24.9	24.8
Minimum Age	21.0	21.0	21.0	21.0
Maximum Age	30.0	30.0	30.0	30.0
Age 30–40 years *n* = 4210				
Mean Age	35.4	35.3	35.7	35.3
Minimum Age	31.0	31.0	31.0	31.0
Maximum Age	40.0	40.0	40.0	40.0
Age 40–50 years *n* = 3939				
Mean Age	45.2	45.2	45.6	45.1
Minimum Age	41.0	41.0	41.0	41.0
Maximum Age	50.0	50.0	50.0	50.0
Age 50–60 years *n* = 3499				
Mean Age	55.1	55.7	55.3	55.1
Minimum Age	51.0	51.0	51.0	51.0
Maximum Age	60.0	60.0	60.0	60.0
Age 60–70 years *n* = 1.196				
Mean Age	65.0	65.3	65.0	65.2
Minimum Age	61.0	61.0	61.0	61.0
Maximum Age	70.0	70.0	70.0	70.0
Age 70–80 years *n* = 1063				
Mean Age	75.1	75.5	74.2	74.5
Minimum Age	71.0	71.0	71.0	71.0
Maximum Age	80.0	80.0	80.0	80.0
Age 80–90 years *n* = 167				
Mean Age	83.6	84.0	84.5	84.4
Minimum Age	81.0	81.0	81.0	81.0
Maximum Age	88.0	90.0	90.0	89.0
Age 90–100 *n* = 35				
Mean Age	92.0	95.5	91.4	94.4
Minimum Age	92.0	92.0	91.0	91.0
Maximum Age	92.0	99.0	94.0	99.0

**Table 3:** Comparison of maximum pupil diameter by gender, concussion, and symptom history.

	Gender							
	Female				Male			
	Concussion				Concussion			
	No Concussion		Concussion		No Concussion		Concussion	
	Symptoms		Symptoms		Symptoms		Symptoms	
	No	Yes	No	Yes	No	Yes	No	Yes
Frequency	1595	5730	1079	3449	1080	4641	733	2325
Percent	7.73%	27.77%	5.23%	16.72%	5.23%	22.49%	3.55%	11.27%
Mean	5.1	5.1	4.8	5	5	5.2	4.7	4.9
*p*-value	0.0078	0.0000	0.0000	0.0000	0.3124	0.0000	0.0000	0.0000
SD	1.2	1.1	0.8	0.1	1.1	1.1	0.8	0.9
Std. error	0.0294	0.0149	0.0252	0.0170	0.0348	0.0164	0.0290	0.0197
*t* statistic	2.6652	5.0464	−8.8316	−6.3487	−1.0107	6.7152	−13.1197	−8.9964

**Table 4:** Comparison of minimum pupil diameter by gender, concussion, and symptom history.

	Gender							
	Female				Male			
	Concussion				Concussion			
	No Concussion		Concussion		No Concussion		Concussion	
	Symptoms		Symptoms		Symptoms		Symptoms	
	No	Yes	No	Yes	No	Yes	No	Yes
Frequency	1595	5730	1079	3449	1080	4641	733	2325
Percent	8%	28%	5%	17%	5%	22%	4%	11%
Mean	3.6	3.6	3.8	3.8	3.5	3.6	3.8	3.8
*p*-value	0.0000	0.0000	0.0000	0.0000	0.0000	0.0000	0.0000	0.0000
SD	0.6	0.7	0.6	0.7	0.6	0.6	0.6	0.7
Std. error	0.015910	0.009060	0.019120	0.011760	0.019163	0.009396	0.022638	0.014176
*t* statistic	−1885.54	−3304.92	−1554.00	−2528.32	−1567.7	−3189.35	−1315.20	−2096.24

**Table 5:** Statistical Tables for Non-Concussion and Concussion Subjects 75% PRT.

Eye75%PRT	Obs	Mean	SE	SD	95% CI	*p*	*t*
**75% PRT Right Eye**							
R No Concussion	3870	3.0758	0.03605	2.2425	3.005159 3.146507	0.0455	2.0001
R Concussion	991	2.6208	0.07098	2.2346	2.481516 2.760109		
Combined	4861	2.9831	0.0322	2.2481	2.919854 3.046284		
diff		0.45502	0.07978		0.2986168. 6114247		
**75% PRT Left Eye**							
L No Concussion	3211	3.1194	0.04124	2.3370	3.038555 3.200283	0.0000	5.9314
L Concussion	991	2.6208	0.07098	2.2346	2.481516 2.760109		
Combined	4202	3.0018	0.03583	2.3227	2.931579 3.072076		
diff		0.49861	0.08406		0.3337997 0.6634134		
**75% PRT Right Eye Compared to Left Eye No Concussion**							
R Eye	3870	3.0758	0.03605	2.2425	3.005159 3.146507	0.4244	−0.7988
L Eye	3211	3.1194	0.04124	2.3370	3.038555 3.200283		
Combined	7081	3.0955	0.02716	2.2858	3.042349 3.148847		
diff		−0.04359	0.05456		−0.1505509 0.0633793		
**75% PRT Both Eyes Male Compared to Both Eyes Female No Concussion**							
Both Eyes Male	2806	3.0944	0.04270	2.2616	3.010699 3.178135	0.7045	−0.3793
Both Eye Female	4145	3.1157	0.03596	2.3152	3.045183 3.186185		
Combined	6951	3.1071	0.02751	2.2936	3.053171 3.161027		
diff		−0.02126	0.05607		−0.1311883 0.0886538		
**75% PRT Both Eyes Male Compared to Both Eyes Female Concussion**							
Both Eyes Male	961.0	2.6433	0.07359	2.2813	2.498927 2.787757	0.0107	−2.5544
Both Eye Female	1138	2.8956	0.06615	2.2315	2.765838 3.025411		
Combined	2099	2.7801	0.04927	2.2574	2.683494 2.876747		
diff		−0.25228	0.09877		−0.445971 0.0585938		
**75% PRT Both Eyes Concussion Symptoms**							
Symptoms	1277	2.9883	0.06276	2.2428	2.865187 3.111441	0.0000	5.3005
No Symptoms	822.0	2.4567	782393	2.2431	2.303113 2.610259		
Combined	2099	2.7801	0.04927	2.2573	2.683494 2.876747		
diff		.53163	0.10030		0.3349343 0.7283214		
**75% PRT Concussion Both eyes vs. Non-Concussion by Male**							
Concussion	961.0	2.6433	0.07359	2.2813	2.498927 2.787757	0.0000	−5.3244
No Concussion	2806	3.0944	0.04270	2.2616	3.010699 3.178135		
Combined	3767	2.9793	0.03706	2.2749	2.906674 3.052012		
diff		−0.45107	0.08471		−0.617174 −0.284975		
**75% PRT Concussion Both eyes vs. Non-Concussion by Female**							
Concussion	1138	2.8956	0.06614	2.2315	2.765838 3.025411	0.0042	−2.8622
No Concussion	4145	3.1157	0.03596	2.3152	3.045183 3.186185		
Combined	5283	3.0683	0.03163	2.2990	3.006275 3.130289		
diff		−0.22006	0.07689		−0.370787 −0.069332		
**75% PRT Concussion Both eyes vs. Non-Concussion Combined Genders**							
Concussion	2099	2.7801	0.04927	2.2573	2.683494 2.876747	0.0000	−5.5692
No Concussion	7081	3.0956	0.02716	2.2858	3.042349 3.148847		
Combined	9180	3.0235	0.02383	2.2831	2.976755 3.070173		
diff		−0.31547	0.05665		−0.426517 −0.204437		

**Table 6:** Statistical Tables for Non-Concussion and Concussion Subjects MCV.

MCV	Obs	Mean	SE	SD	95% CI	*p*	*t*
**MCV Concussion by Female**							
Concussion	2767	4.88963	0.008990	4.7288	4.713358 5.065904	0.2326	−1.1938
No Concussion	12,803	4.75808	0.004740	5.3634	4.665163 4.850988		
Combined	15,570	4.78146	0.004212	5.2563	4.698885 4.864024		
diff		−0.1316	0.011019		−0.347549 −0.084439		
**MCV Concussion by Male**							
Concussion	2232	4.87452	0.010466	4.9447	4.669271 5.079769	0.0442	−2.0127
No Concussion	10,044	4.63931	0.004994	5.0049	4.541414 4.737195		
Combined	12,276	4.68207	0.004508	4.9946	4.593709 4.770433		
diff		−0.23522	0.011686		−0.464285 −0.006146		
**MCV Concussion vs. Non-Concussion**							
Concussion	4999	4.88128	0.006822	4.8250	4.747533 5.015023	0.0062	−2.7378
No Concussion	22,847	4.66249	0.003379	5.1985	4.596268 4.728719		
Combined	32,846	4.70066	0.003033	5.1359	4.64121 4.760105		
diff		−0.21878	0.007991		−0.375420 −0.062150		
**MCV Concussion Symptoms**							
Symptoms	1685.1	4.87697	155027	4.7412	4.650426 5.103514	0.9641	−0.0450
No Symptoms	3317.0	4.88347	845178	4.8677	4.717755 0.049178		
Combined	5002.0	4.88128	682218	4.8250	4.747533 5.015023		
diff		−0.00650	0.14436		−2894987 0.2765072		

**Table 7:** Latency of the Pupillary Light Response.

	Gender							
	Female				Male			
	Concussion				Concussion			
	No Concussion		Concussion		No Concussion		Concussion	
	Symptoms		Symptoms		Symptoms		Symptoms	
	No	Yes	No	Yes	No	Yes	No	Yes
Frequency	1595	5730	1079	3449	1080	4641	733	2325
Percent	7.73%	27.77%	5.23%	16.72%	5.23%	22.49%	3.55%	11.27%
Mean	0.2068	0.2061	0.1979	0.1974	0.2034	0.2066	0.1958	0.1964
SD	0.0567	0.0563	0.0554	0.0560	0.0586	0.0583	0.0589	0.0565

## Data Availability

Not applicable.
